# Consistency of parent-report SLC6A1 data in Simons Searchlight with Provider-Based Publications

**DOI:** 10.1186/s11689-022-09449-7

**Published:** 2022-06-28

**Authors:** Jennifer M. Bain, LeeAnne Green Snyder, Katherine L. Helbig, Dominique D. Cooper, Wendy K. Chung, Kimberly Goodspeed

**Affiliations:** 1 Department of Neurology, Division of Child Neurology, Columbia University Irving Medical Center, New York, NY USA; 2 Simons Foundation, New York, NY USA; 3 Department of Pediatrics, Division of Neurology and Epilepsy NeuroGenetics Initiative, Children’s Hospital of Philadelphia, Philadelphia, PA USA; 4 University of Texas Southwestern Medical Center, Dallas, TX USA; 5 Department of Pediatrics, Columbia University Irving Medical Center, New York, NY USA; 6 Department of Pediatrics, Division of Neurology, University of Texas Southwestern Medical Center, Dallas, TX USA

**Keywords:** SLC6A1, Neurodevelopmental disorder, Intellectual disability, Genetic, Epilepsy, Autism, Hypotonia, Movement disorder

## Abstract

**Background:**

*SLC6A1*-related disorder is a recently identified, rare, genetic neurodevelopmental disorder that is associated with loss-of-function variants in SLC6A1. This gene encodes GABA transporter type I that is responsible for re-uptake of GABA from the synapse into the pre-synaptic terminal or circulating neuroglia. Based upon retrospective review of published cases and available research databases including Epi25 collective and SLC6A1 Connect patient database, the phenotypic spectrum is broad and includes developmental delay, epilepsy, and autism or autistic traits. *SLC6A1* is one of the genes included in the Simons Searchlight registry, which includes standardized data collection across genetically identified neurodevelopmental conditions.

**Methods:**

In this study, we compare parent-report measures of phenotypic features in the Simons Searchlight registry to previously published, provider-reported cases to assess if parent-report measures are consistent with what has been reported in the literature.

**Results:**

There were 116 participants in the provider-reported dataset compared to 43 individuals in the caregiver-reported dataset. Carriers in Searchlight had 83 unique pathogenic or likely pathogenic variants in SLC6A1, which were predominantly missense or nonsense variants. There was no significant difference between groups for the prevalence of developmental delay, ASD, or ADHD. Caregivers more often reported hypotonia, while epilepsy was slightly more frequently reported by providers.

**Conclusions:**

We propose that standardized parent-report data collection methods are consistent with provider reports on many core features of SLC6A1-related disorder. The availability of patient registries and standardized natural history studies may fill an important need in clinical trial readiness programs, with larger sample sizes than smaller published case series.

**Supplementary Information:**

The online version contains supplementary material available at 10.1186/s11689-022-09449-7.

## Background

*SLC6A1*-related disorder (SRD) is a newly identified cause of developmental and epileptic encephalopathies and consistently appears among the common genetic causes of autism spectrum disorder and neurodevelopmental disability [1–6]. The SLC6A1 gene is located on the short arm of chromosome 3 (GRCh38 genomic coordinates: 3:10,992,733–11,039,248) [7]. It encodes the GABA transporter type 1 (GAT1), which mediates the reuptake of GABA into the presynaptic terminal of neurons and glia [7]. Loss of function variants in SLC6A1 have been associated with a spectrum of neurodevelopmental disorders, including autism spectrum disorders (ASD), developmental delay and intellectual disability (ID), and a variety of seizure types including absence seizures and myoclonic-atonic seizures [1–6]. Currently, therapeutic treatments aim to reduce disease burden by managing the symptoms and manifestations of this condition. Larger natural history studies are essential for recruitment into precision medicine clinical trials and as comparison arms for such studies. For rare conditions, such as SRD, it can be difficult to enroll individuals around the world in research for a variety of logistical barriers, and the available literature largely consists of clinician-reported case series. These reports can vary greatly in data collection methodologies and may focus on facets of the disorder depending on the journal in which they are published and the main interests of the publishing researchers. Moreover, there remains difficulty in identifying a true prevalence with overlapping case series and no universally accepted identifier. With emerging therapies in genetically identified neurodevelopmental disorders, it is important for there to be a concerted effort towards standardization of rigorous longitudinal data collection and interpretation. Due to their level of developmental disability, seizure burden, as well as other medical and financial barriers, it can be difficult for patients with genetic neurodevelopmental disorders to travel to clinical sites to participate in natural history studies that are critical to the success of future clinical trials. The Simons Searchlight study reduces the patient burden through a standardized longitudinal remote collection of data from parents and caregivers from around the world for research into rare genetic disorders [8].

Our respective research groups individually published on the *SLC6A1* cohort, first using a clinician-referred case series sample [3] and more recently incorporating the data collected from parents and caregivers enrolled in Simons Searchlight [4]. The clinician-referred case series compiled 116 cases with *SLC6A1* variants from previously published case series, Epi25 collective, and SLC6A1 Connect database. This study included a clinical report of phenotypes from medically trained professionals. We showed the most common clinical features included epilepsy (92/101, 91.1%), developmental delay and cognitive impairment (46/56, 82.1%), and autistic traits (20/92, 22.8%). We also showed that providers reported that nearly all subjects demonstrate developmental delay (46/55, 83.6%) after seizure onset, and we had detailed seizure semiology on 56 of the 92 subjects with epilepsy [3]. Simons Searchlight, under the Simons Foundation Autism Research Initiative (SFARI), allows families to provide their data to a research database from home through phone interviews and online surveys. The Simons Searchlight program enrolls individuals with genetic disorders associated with a neurodevelopmental disorder, including autism, or neurodegenerative disease [8]. We were interested in assessing whether the parent-report method of data collection in Simons Searchlight was comparable to provider-published data, by comparing the cross-sectional prevalence of key clinical features across both studies. In this study, we will describe and compare the provider- and caregiver-reported data to assess their consistency and determine whether caregiver-reported data are an accurate tool in understanding the phenotypic spectrum of *SLC6A1*-related disorder.

## Methods

We used two datasets of SLC6A1 subjects: the provider-reported dataset (Literature) and the caregiver-reported dataset (Searchlight). The provider-reported data (Literature) includes the prevalence of neurological features of SRD from published cases, Epi25 Collaborative Database, and the SLC6A1 Connect Foundation. Data extracted from the Epi25 Collaborative Database included exclusively genotype and International League Against Epilepsy (ILAE) categorization. SLC6A1 Connect Foundation compiled clinical data reported by each subject’s referring physicians. The caregiver-reported dataset (Searchlight) is the Simons Searchlight SLC6A1 Registry. Simons Searchlight collects medical and developmental history via interviews with genetic counselors, as well as behavioral information by electronic surveys and standardized questionnaires. Genetic test reports submitted to Simons Searchlight are reviewed by a single central team of board-certified genetic counselors and laboratory geneticists who verify that all reported results are pathogenic/likely pathogenic, consistent with American College of Medical Genetics guidelines [9, 10]. We used the Version 8 dataset from the Simons Searchlight Database for this data analysis. We looked at variants common to each group and attempted to match subjects by gender. The age was not used because the recorded age in each dataset was the age at enrollment or inclusion in the study, which could differ between datasets. Due to missing gender data within the provider-reported dataset on many of the potential duplicates, we were unable to confidently match individual subjects between the two groups. Due to our inability to accurately match overlapping subjects, we report the percentage of unique variants within each group as a proxy for unique individuals.

### Group-level comparison of prevalence of clinical features

We reviewed de-identified clinical and genotypic information and determined the mean, frequency, and standard deviation of each reported clinical feature. The prevalence of previously identified key clinical features including (1) epilepsy, (2) intellectual disability or developmental delay (ID/DD), (3) autism spectrum disorder (ASD), (4) hypotonia, (5) movement disorders or ataxia, and (6) attention deficit and hyperactivity disorder (ADHD) were calculated in both datasets. Only clinical features which were explicitly reported as being present or absent were included in the analysis.

### Comparison of reported ASD/ID/IDD to standardized measures

The Simons Searchlight database included quantitative measures to assess for ASD traits. The Social Communication Questionnaire-Lifetime (SCQ) and Social Responsiveness Scale-2 (SRS-2) assess for social-communication skills, repetitive behaviors, and other associated behaviors. The Simons Searchlight database also includes a quantitative measure of global adaptive functioning using the Vineland Adaptive Behavior Scale- 2nd edition (VABS). We compared participants’ results on these assessments to the parental report of ASD to assess for differences in adaptive functioning between those with and without reported ASD as well as to assess for consistency between reported ASD and scores on the SCQ and SRS-2. One participant with an Adaptive Behavior Composite score of < 40 on the VABS was excluded from the analysis because the SCQ and SRS do not accurately capture symptoms of ASD for individuals with adaptive functioning below this cutoff.

### Statistical analysis

All statistical analysis were performed using Statistical Analysis Software (SAS, 9.4). Comparisons were made using chi-square tests. Where assumptions were not met (i.e., prevalence < 5), Fisher’s exact test was used. Descriptive statistics were calculated for the SRS-2, SCQ, VABS, and of age of developmental milestone attainment. *T* tests were used to assess the difference in mean scores on the SCQ and SRS-2 for those with and without ASD by parental report. The Wilcoxon-matched pairs sign rank-sum test was used to assess the difference in chronological age and estimated age equivalents on all subdomains of the VABS. All statistical tests used a two-tailed hypothesis and a *p* value of < 0.05 was considered statistically significant.

## Results

There were 116 participants in the provider-reported dataset (Literature), who were previously described [4]. The caregiver-reported dataset (Searchlight) enrolled 43 individuals with pathogenic or likely pathogenic variants in *SLC6A1* at the time of data analysis. Sex was available on half of the individuals in the provider-reported group, with 26 males and 32 females. There were 22 males and 21 females in the caregiver-reported cohort (Searchlight). Ages ranged from 16 to 336 months (mean 125, SD 74) in the provider-reported group (Literature) and 7 to 280 months (mean 97, SD 71) in the caregiver-reported group (Searchlight). Between the two datasets, there were 83 unique molecular variants identified, predominantly missense or nonsense variants, all of which were pathogenic or predicted pathogenic by ACMG criteria (Supplemental Table 1). Of the 83 unique variants, 15 variants (17%) were present in both datasets, with concern for being an overlapping subject. There were 24 variants noted only in the parent-report sample and 48 variants noted only in the provider-based cases. We compared the prevalence of core phenotypic features between samples using chi-square tests. A Fisher’s exact test was used to compare the rate of ADHD between the two samples due to a lower number of observations for this condition. There was no significant difference between samples for the prevalence of developmental delay, ASD, or ADHD. There was a statistically significant difference in hypotonia (*p* < 0.0001), with higher prevalence in the caregiver-reported (Searchlight) dataset as compared to the provider-reported (Literature) dataset. Epilepsy was slightly more frequent in the provider-reported (Literature) cohort, and movement disorders were slightly more frequent in the caregiver-reported (Searchlight) cohort; however, neither reached significance (Table 1).

**Table 1:** Subject demographics and prevalence of core clinical features of *SLC6A1*-related disorder between the caregiver-reported (Searchlight) data and the provider-reported (Literature) data

		**Searchlight (*n* = 43)**	**Literature** **(total, *n* = 116)** **(gender, *n* = 58)** **(age, *n* = 40)**	***p value***
**Demographics**	Gender (M to F)	22:21	26:32	
	Age, mean (SD), range	97 months, 71, 7 to 280 months	125, 74, 16 to 336 months	
**Clinical characteristics**		*n* = 35	*n* = 116	
	Developmental delay or intellectual disability	29/35 (82.9)	51/55 (92.7)	0.15
	Epilepsy	28/35 (80)	92/101 (91.1)	0.08
	Autism spectrum disorder (ASD)	11/35 (31.4)	21/92 (22.8)	0.36
	Attention deficit hyperactivity disorder (ADHD)	4/35 (11.4)	9/47 (19.2)	0.38
	Hypotonia	24/35 (68.6)	8/46 (17.4)	< 0.0001
	Movement disorder	17/35 (48.6)	13/46 (28.3)	0.07

**Fig. 1:**
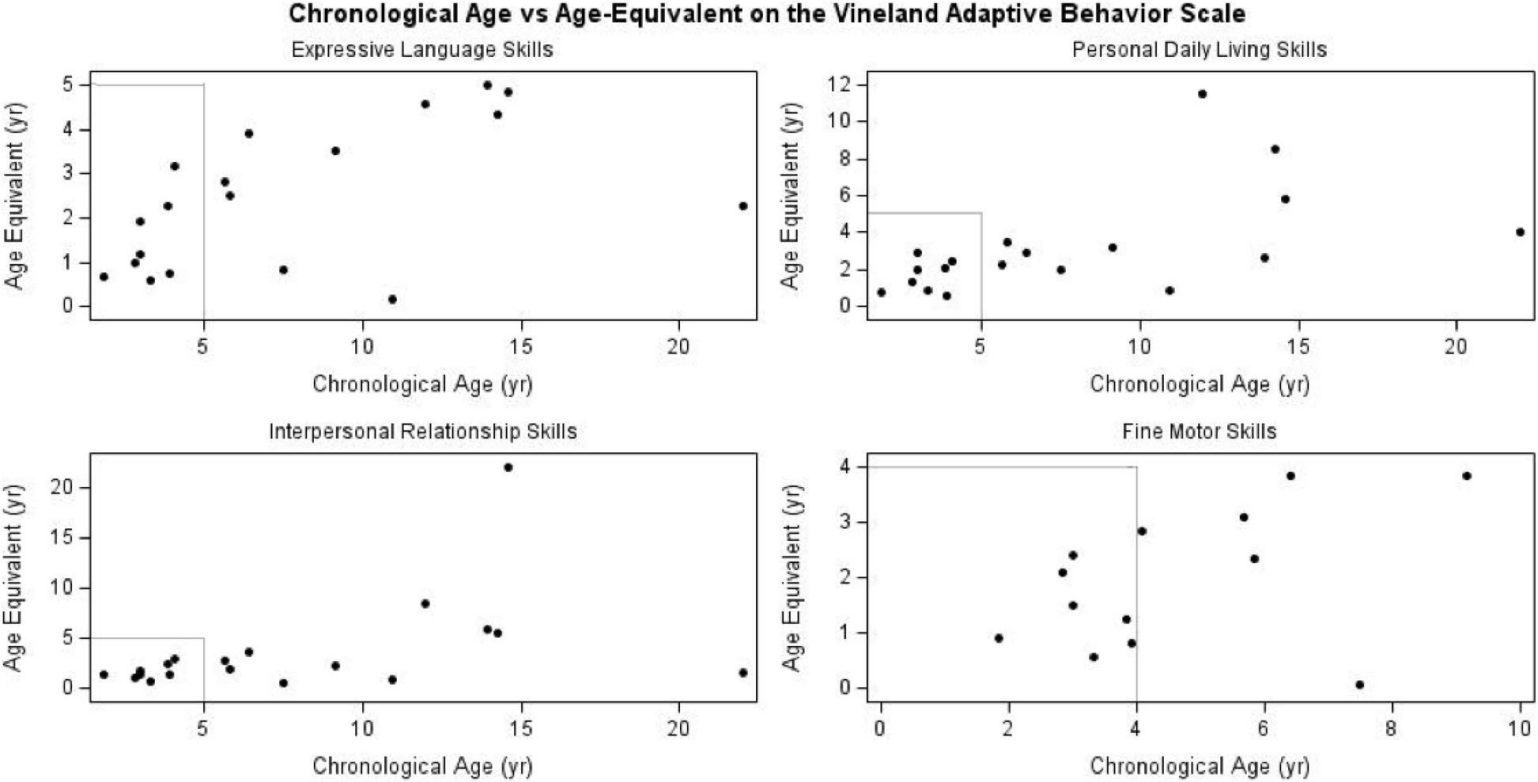
The Vineland Adaptive Behavior Scale (VABS) is a standardized assessment of adaptive behavior skills across four domains: Communication, Motor, Daily Living Skills, and Socialization. The chronological age at the time of evaluation (years) is plotted against the estimated age-equivalent of the following subdomains: expressive language skills, personal daily living skills, interpersonal relationship skills, and fine motor skills. The motor domains of the VABS have a ceiling of approximately 8 years. The gray lines represent the cross-section of the chronological age and age-equivalent at 5 years for expressive language skills, personal daily living skills, and interpersonal relationship skills, and 4 years for fine motor skills

Of the 43 individuals enrolled in the Simons Searchlight SLC6A1 Registry, standardized questionnaires were available on a subset of subjects: Vineland Adaptive Behavior Scale-II (VABS, *n* = 19), Social Responsiveness Scale-2 (SRS-2, *n* = 19), and Social Communication Questionnaire-Lifetime (SCQ, *n* = 24). Of the 24 individuals who completed the SCQ, two were missing data on their history of ASD diagnosis. The mean VABS Adaptive Behavior Composite score for the group was 64.5 (SD 15.9), which is approximately 2.5 SD below tshe mean of 100. There was no difference in mean subdomain scores on the VABS between those with or without reported ASD (Table 2). Age-equivalent data were examined qualitatively by age. When comparing the chronological age of the patient at the time of evaluation to their estimated developmental age equivalent using the Wilcoxon sign rank test, age equivalents were significantly lower than current age across all subdomains (*p* < 0.005, see Fig. 1). Expressive language was noted to be modestly higher at older ages, toward attainment of approximately the 4–5-year-old level for most children (indicating the presence of at least phrase speech or short sentences) (Fig. 1). Qualitatively, there is evidence that several children are learning to read or are able to read. Gross and fine motor levels show a plateau at the 2–4-year-old level in the small group of older children (within a measurement ceiling of 84 months), indicating that most children have learned to walk and have mobility. Daily living and social skills show greater variability in attainment, up to the 8–12-year-old level for several individuals, at older ages.

**Table 2:** A summary of the standard scores on each domain of the Vineland Adaptive Behavior Scale (VABS) in Simons Searchlight (*n* = 19) and comparison of scores between those with parent-reported autism (*n* = 5) and those without autism (*n* = 14). A comparison of the Social Responsiveness Scale-2 (SRS-2, *n* = 19) and the Social Communication Questionnaire-Lifetime (SCQ, *n* = 24) scores among those with reported ASD and those without reported ASD (No ASD) in Simons Searchlight

	**All**	**ASD**	**No ASD**	***p value*** **(ASD vs no ASD)**
**VABS**	*n* = 19	*n* = 5	*n* = 14 (*n* = 7 for motor)	
**Adaptive composite** **Mean (SD)**	64.5 (15.9)	64.6 (9.5)	64.5 (17.9)	0.99
**Communication** **Mean (SD)**	62.6 (14.7)	65.8 (9.4)	61.4 (16.3)	0.58
**Daily living skills** **Mean (SD)**	67.1 (16.3)	68.2 (15.7)	66.7 (17)	0.87
**Socialization** **Mean (SD)**	69.2 (16)	68.8 (11)	69.3 (17.8)	0.96
**Motor skills** **Mean (SD)**	65 (9.76)	64.4 (10.7)	65.4 (9.7)	0.87
	SRS, *n* = 19 SCQ, *n* = 24	SRS, *n* = 7 SCQ, *n* = 7	SRS, *n* = 12 SCQ, *n* = 15	
**SRS** **Mean (SD)**	73.3 (9.9)	74.3 (10.3)	71.8 (9.8)	0.6
**SCQ** **Mean (SD)**	14.2 (7.5)	14.1 (5.6)	13 (7.9)	0.73

On standardized questionnaires to assess autistic traits, individuals in Simons Searchlight had a mean T score of 73.3 (SD 9.9) on the SRS-2 (*n* = 19) and a mean total score of 14.2 (SD 7.5) on the SCQ (*n* = 24) (Table 2). T scores on the SRS-2 ranged from 59 to 90 with most falling in the moderate (66 to 75) to severe range (greater than 76) as a group on the SRS-2. On the SCQ, scores ranged from 2 to 28 with approximately half falling above the cut-off of 15, a quarter of which were greater than 22. When using a more conservative cut-off score of 22 on the SCQ and 76 on the SRS-2, there was 50% and 63% agreement between the parental report of ASD and scores on the standardized measures. Both instances of elevated scores without a parental report of ASD and instances of low scores with a parental report of ASD were seen. There were five individuals who had scores above the cut-off range on the SRS-2, SCQ, or both measures who did not have a history of ASD, and eight who had a history of ASD reported by caregivers but scores below the cut-offs for ASD. Comparisons using *t* tests found no differences in group mean SCQ and SRS-2 scores among those with or without a reported diagnosis of ASD (Table 2).

In the caregiver-report (Searchlight) group, early development showed a very wide range, inclusive of individuals who achieved developmental milestones within normal limits (Table 3). However, mean ages of attainment were in the delayed range for the group, and inspection of the data revealed that 50% of individuals were delayed in first words (> 18 months), 75% were delayed in phrase speech (> 24 months), and 45% were delayed in walking (> 16 months).

**Table 3:** Age of attainment of developmental milestones among children 16 months to 23 years in Simons Searchlight. *1 unknown age reported as > 84 months was excluded

VABS	All	ASD	No ASD	*p value* (ASD vs no ASD)
	*n* = 19	*n* = 5	*n* = 14 (*n* = 7 for motor)	
**Adaptive composite** **Mean (SD)**	64.5 (15.9)	64.6 (9.5)	64.5 (17.9)	0.99
**Communication** **Mean (SD)**	62.6 (14.7)	65.8 (9.4)	61.4 (16.3)	0.58
**Daily living skills** **Mean (SD)**	67.1 (16.3)	68.2 (15.7)	66.7 (17)	0.87
**Socialization** **Mean (SD)**	69.2 (16)	68.8 (11)	69.3 (17.8)	0.96
**Motor skills** **Mean (SD)**	65 (9.76)	64.4 (10.7)	65.4 (9.7)	0.87
	SRS, *n* = 19 SCQ, *n* = 24	SRS, *n* = 7 SCQ, *n* = 7	SRS, *n* = 12 SCQ, *n* = 15	
**SRS** **Mean (SD)**	73.3 (9.9)	74.3 (10.3)	71.8 (9.8)	0.6
**SCQ** **Mean (SD)**	14.2 (7.5)	14.1 (5.6)	13 (7.9)	0.73

## Discussion

To our knowledge, this is the first study to compare the prevalence of core clinical features of a single rare genetic disorder, in this case SLC6A1, found in databases generated by medical professionals and content experts to data in a caregiver-reported patient registry, Simons Searchlight. With any retrospective data review, investigators are limited by the structure of existing datasets. As demonstrated in our large retrospective provider-reported dataset, detailed clinical phenotyping data are limited [4]. In that cohort, clinical data were available on the majority of the group for conditions including epilepsy, developmental delay, and autism spectrum disorder; however, less than half had data available on common comorbid conditions such as ADHD, hypotonia, and movement disorders. In addition to missing data elements, the depth of data was also limited. Qualitative reports of broad diagnoses such as developmental delay are provided rather than detailed standardized testing information or categorization of severity of deficits. To simplify analysis of the retrospective data, we combined developmental delay and intellectual disability. This is a major limitation of retrospective data collection because not all who have developmental delay will also meet criteria for intellectual disability and intellectual disability may be a confounding variable. Standardized validated measures like the Vineland Adaptive Behavior Scales would allow for more precise quantitative data to be collected from individuals, which may highlight a paradigm shift in the traditional in-person evaluations for rare disorders to utilizing a combination of in person and virtual assessments [11]. In addition, medical evaluations may consider including standardized measures such as the Vineland to collect valuable data that also can be easily translated across various clinical sites.

Moreover, it becomes difficult to assess whether there are overlapping subjects being reported in larger case series by different investigators without the incorporation of a common identifier such as the global unique identifier (GUID). Utilization of the GUID across more studies in rare disease, as is done in Simons Searchlight, could allow for matching subjects between studies, which would increase the portability and potentially increase the power of clinical datasets in rare disease populations. Given the rarity of SRD, it is plausible that a single individual is included in more than one database, and with insufficient phenotypic and demographic data, it is difficult to rule out the presence of overlapping patients within these datasets, especially if there are recurring genotypes. If individuals are duplicated within the combined provider-reported dataset, there may be an inappropriate weighting of their associated clinical features, skewing the data. In addition, these datasets are often retrospective cohort studies, and there is a lack of prospective or longitudinal data collection.

We sought to determine if caregiver report data from a relatively large registry, Simons Searchlight, would show consistency with what has previously been reported in the literature based on medical provider data. Simons Searchlight verifies genetic testing results to confirm pathogenicity of the variants, collects medical records, and also assigns unique identifiers to avoid duplication of subjects. Importantly, they collect data prospectively using validated questionnaires that are commonly used in clinical practice and other research studies and provide more behavioral phenotypic information. Though the caregiver-reported dataset (Simons Searchlight) is a smaller sample, data collection is more rigorous and standardized, and there are fewer missing data points. In the case of the *SLC6A1* cohort of Simons Searchlight, the sample size is still small and only a single time-point is available. As additional patients are identified and multiple time-points are collected longitudinally, the power of this registry will increase. Even still, neither of these observational study designs are sufficient to supplant a prospective natural history study, but rather, provide insights on the spectrum of a rare disease and inform the design of prospective studies by collecting an unbiased, structured dataset on all individuals.

The concordance of the prevalence of epilepsy and autism spectrum disorder between the two groups supports that these are two of the core clinical features of SRD. Further, there were the fewest number of missing data points for these two clinical features in the provider-reported dataset. Findings on the seizure survey and on standardized ASD instruments within the Simons Searchlight patient registry support the presence of epilepsy and of autistic traits such as repetitive behaviors, though clinical diagnoses of ASD were discordant with scores on ASD instruments. There was no difference in scores between those with ASD and those without ASD. However, questionnaire screeners are notoriously limited in their ability to distinguish ASD in the presence of intellectual disability and other comorbidities. Further clinician-generated evaluations, such as those including the Autism Diagnostic Observation Scale in individuals of sufficient mental age, are needed to evaluate whether individuals with SRD have ASD or if these ASD screening tools are showing elevated scores for autistic traits, such as repetitive behaviors. The relatively higher reports of DD/ID per caregiver report are also reflected in both early and late developmental delays according to developmental milestones and results of the standardized Vineland interview.

In contrast, there was higher prevalence of hypotonia and movement disorders in the caregiver-reported dataset, with hypotonia reaching statistical significance. The high rate of missing data points within these clinical features in the provider-reported data highlights a limitation of the provider-reported dataset, in which the phenotypic descriptions are not standardized or clinically confirmed. Further, published case reports often highlight certain features of a disorder while omitting others that are relevant to the audience of the journal in which the publication appears. A few potential explanations include (1) Simons Searchlight, because of the smaller sample, is subject to sampling bias; (2) these features were present in the provider-reported data but not included in the publication or database; or (3) these features were omitted from the provider-reported data because they were not present in individuals. Because SRD was initially described in epilepsy journals, many of the early descriptions of the disorder included deep phenotyping of the seizure semiology and developmental abilities, which are a significant source of morbidity in developmental epileptic encephalopathies. While features such as hypotonia are quite common among neurodevelopmental disorders, the reports in the literature may omit these details. As the phenotypic spectrum of genetic developmental epileptic encephalopathies expands, additional features, such as movement disorders like tremor or stereotypy, are emerging as a relatively common feature of these disorders. Patient registries with standardized assessment tools applied to the entire sample have the potential to uncover new features of disorders or identify clinical outcome measures that could inform a future clinical trial. Finally, harmonization of tools across patient registries and agreement upon a set of common data elements and unique common identifiers would facilitate the compilation of multiple datasets, improve portability of clinical data, and potentially expedite important discoveries that could make rare disease clinical research more efficient.

## Conclusion

Observational studies are critical to establishing the core clinical features of disorders and exploring potential clinical outcome measures that capture the spectrum of severity among affected individuals. Retrospective reviews of medical charts, published literature, and available databases are a valuable resource, but there are limitations in terms of inconsistent data collection methods. Comparatively, patient registries with prospective, standardized data collection tools, such as Simons Searchlight, increase the power of the data by reducing the number of missing data points and allowing for comparison of clinical features between different disorders within the same registry. Future directions would include greater harmonization among disparate clinical databases such that a set of minimum common data elements are collected on a broad range of studies and increasing the power and portability of clinical datasets.

## Supplementary Information

**Additional file 1:** **Table S1.** ACMG criteria.

## Data Availability

Anonymized data will be shared by request from any qualified investigator. For those participants who have consented to research with Simons Searchlight, data are available through SFARIbase (base.sfari.org/).
